# Molecular characterization of multi-drug-resistant *Staphylococcus aureus* in mastitis bovine milk from a dairy farm in Anhui, China

**DOI:** 10.3389/fvets.2022.966533

**Published:** 2022-08-22

**Authors:** Junjun Liu, Xin Wang, Chongliang Bi, Khalid Mehmood, Farah Ali, Jianhua Qin, Zhaoqing Han

**Affiliations:** ^1^College of Veterinary Medicine/Traditional Chinese Veterinary Medicine, Hebei Agriculture University, Baoding, China; ^2^College of Agriculture and Forestry, Linyi University, Linyi City, China; ^3^Faculty of Veterinary and Animal Sciences, The Islamia University of Bahawalpur, Bahawalpur, Pakistan

**Keywords:** *Staphylococcus aureus*, mastitis, milk, PCR, bovine

## Abstract

Mastitis is an economically important disease in the dairy industry, which is caused by various infectious pathogens. There is limited information known about the situation of drug resistance and virulence factors of *Staphylococcus aureus* (*S. aureus*) in mastitis bovine milk in Anhui. Therefore, a total of 125 fresh milk samples from clinically mastitis-positive bovine animals were collected. The bacteria pathogens were identified *via* bacterial culture, Gram staining, biochemical analysis, DNA extraction, 16s rRNA amplification, and phylogenetic analysis. Drug resistance analyses were performed through drug-resistant genes and virulence genes amplification. Results showed that a total of 24.8% (31/125) bacterial isolates were isolated and identified as *S. aureus* by Gram straining, biochemical reactions, and 16 s rRNA genes blasting. Multiple sequence alignment analysis found that the current isolates were highly similar (96.9–100.0%) to previous isolates. Phylogenetic analysis demonstrated that *S. aureus* was similar with MK809241.1 isolated from food in China and wCP030426.1 isolated from a person in the United States. The bacterial isolates were detected resistant to 11 antibiotics, such as Penicillin G, SXT, Ciprofloxacin, Norfloxacin, Polymyxin B, Levofloxacin, Chloramphenicol, Clindamycin, Clarithromycin, Erythromycin, and Spectinomycin. Drug-resistant genes of blaZ, ermC, rpoB, and ant (4')-la were successfully amplified. Virulence genes of hla, nuc, clfa, and eta were found in *S. aureus* bacteria. The current study isolated *S. aureus* from milk samples and revealed its drug-resistant situation, drug-resistant genes, and virulence genes. Hence, regular monitoring of *S. aureus* in milk samples from dairy cows may contribute to the prevention and treatment of public health concerns causing bacteria in this region.

## Introduction

Mastitis is the inflammation of the udder due to the infection of a variety of bacteria that enter the mammary glands and damage them (1). Mastitis is a serious livestock disease, which usually causes significant economic losses along with clinical and sub-clinical symptoms in highly productive dairy animals (2). The opportunistic pathogen *Staphylococcus aureus* (*S. aureus*) is a representative microflora species, which is commonly disseminated on healthy skin and mucous membranes of people and animals (3–7). Previous studies reported that over 50 species of *Staphylococcus* could lead to mastitis, among them *S. aureus* is generally recognized as one of the most frequently examined pathogens in cattle (8). Moreover, it is causing udder inflammation in cows, which leads to infections or intoxications to consumers (9, 10). In Asia, *S. aureus* is considered the major etiologic pathogen of mastitis in cattle and buffaloes (11). The infection of *S. aureus* in dairy cows not only harms the health of animals and milk production or quality (2) but also potentially transmits this pathogen to herdsmen or citizens (9).

Nowadays, various kinds of antibiotics are used for the treatment and control of mastitis in dairy animals. As a result of extensive and irrational use of antibiotics, drug-resistant bacteria are increasing day by day and becoming a major problem for human health worldwide (12–21). The public health concern is that drug-resistant or multi-drug-resistant *S. aureus* strains are occasionally reported to cause serious dermatitis, pneumonia, mastitis, septicemia, myelitis, and bacterial endocarditis (3, 4). Previously, the prevalence of penicillin resistance to *S. aureus* in mastitis cases was more than 50% in the United States of America, Ireland, and England (22). Multi-drug-resistant *S. aureus* strains were also reported in Bangladesh, Jordan, Ethiopia, and the Czech Republic (10, 11, 23, 24).

Anhui province is located in the Yangtze River Delta region in East China, with a northern latitude of 29°41′-34°38′ and an east longitude of 114°54′-119°37′ (Supplementary Figure S1). The warm, temperate, and subtropical climates contribute to the prosperous agricultural development in this region. In 2020, there were 948,000 head of cattle and 376,400 tons of milk produced in the Anhui province (National Bureau of Statistics, https://data.stats.gov.cn/index.htm). A previous study reported that the prevalence of *S. aureus* in dairy cows was 29.1% on farms in this province (25). However, limited information is available about the situation of drug resistance and virulence factors of *S. aureus* in mastitis bovine milk products in Anhui. Herein, this study was carried out to explore the molecular characterization of multi-drug-resistant *S. aureus* in mastitis bovine milk in the Anhui province.

## Materials and methods

### Samples collection

A total of 125 milk samples were aseptically collected from 125 Holstein cows with clinical mastitis in commercial sterile tubes (Thermo Fisher Nunc™, China) by a veterinarian from a dairy farm in 2022 in Anhui, China. Each cow had local signs of inflammation, such as hot, painful, and enlarged mammary glands, which were confirmed by a veterinarian. The collected samples were shipped to a clinical laboratory of Hebei Agricultural University by dry ice (Meijie Dry Ice Technology Co., LTD, Hefei, China) for further processing.

### Bacterial culturing, isolation, and identification

From each milk sample, 0.1 ml raw milk was mixed with 0.9 ml Luria-Bertani (LB) broth (Qingdao Hope Bio-Technology Co., Ltd) in a 5 ml tube and incubated at 36 ± 1°C in an orbital shaker for an h for bacterial enrichment. Then, enriched bacteria were plated on Mannitol salt agar (Qingdao Hope Bio-Technology Co., Ltd) in Petri dishes and incubated at 36 ± 1°C for 24 h. The single colonies were utilized for morphology, Gram staining, and biochemical analysis (Hangzhou Microbial Reagent Co., Ltd). The biochemical reactions, including N-acetyl-glucosamine, trehalose, sucrose, urea, mannose, maltose, xylose, nitrate reduction, N-mannitol, and lactose, were performed for the identification of bacteria.

### DNA extraction, 16s rRNA amplification, and phylogenetic analysis

A single colony was cultivated in a sterile tube with 5 ml of LB broth and incubated at 36 ± 1°C in an orbital shaker for 24 h, then 1 ml medium was taken and centrifuged at 8,000 rpm for 5 min for DNA extraction. The DNA extraction of bacteria was employed through a commercial bacterial genomic DNA extraction kit (Solarbio life sciences, China) as the previous study described (1). Then, the 16s rRNA gene was amplified by using universal primers (27F, Forward, 5'-AGA GTT TGA TCM TGG CTC AG-3', reverse, 1492R, and 5'-TAC GGY TAC CTT GTT ACG ACT T-3'). The 25 μl PCR reaction mixture contains 1 μl DNA template, 1 μl of forward and reverse primers, respectively, 12.5 μl Taq PCR Master Mix (Sangon Biotech, China), and 9.5 μl distilled water. The PCR amplification contained 35 PCR cycles with 95°C for 30 s, 62°C for 35 s, and 72°C for 45 s in each cycle after an initial hot start at 95°C for 3 min and ending with 72°C for 5 min. After that, all the 16s rRNA PCR products were examined through 0.8% agarose gel electrophoresis. Then, the positive 16s rRNA PCR amplified products with the expected size were purified by using the GenElute™ Gel Extraction Kit (Catalog number NA1111, Sigma-Aldrich, The United States of America) according to the manufacturer's explanatory memorandum.

All the purified 16s rRNA PCR samples were further subjected to bidirectional gene sequencing *via* a 3730xl DNA Analyzer at Sangon Biotech (Shanghai, China). Multiple sequence alignments were performed between 16s rRNA of *S. aureus* bovine milk isolates and references genes of 16s rRNA of bacteria available in the NCBI database by piloting Lasergene (Version 7.0). These used reference strains were *S. aureus* strain RM_AST_SA006 (MK809241.1), *S. aureus* strain MRSA-5043 (MT250912.1), *S. aureus* strain 18BWI (KX456106.1), *S. aureus* strain 3-355MR (OK090920.1), *S. aureus* strain DSM 20231 (Type) (MN652637.1), *S. aureus* strain ER04320.3 (CP030426.1), *S. aureus* strain AR_0226 (CP029664.1), *Streptococcus alactolyticus* (LC632504.1), *Streptococcus oricebi* JCM 30719 (LC638727.1), *Streptococcus oralis* 4-KN-2020 (LC545467.1), *Streptococcus mitis* 3-KN-2020 (LC545466.1), *Streptococcus loxodontisalivarius* JCM 19287 (LC589217.1), *Streptococcus troglodytae* JCM 18038 (LC521977.1), *Streptococcus dentasini* JCM 17943 (LC520002.1), *Streptococcus fryi* JCM 16387 (LC519990.1), *Streptococcus sp*. Marseille-P5794 (LR597666.1), *Streptococcus thermophilus* YS5 (LC485981.1), and *Escherichia coli* JCM 16946 (LC682250.1) (out group).

### Phylogenetic analysis of 16s rRNA gene of *S. Aureus* bovine milk isolates *via* MEGA

The phylogenetic relationship analysis between the 16s rRNA gene of *S. aureus* bovine milk isolates and reference bacteria genes was performed by utilizing MEGA (Version 6.0) through neighbor-joining (NJ) methods computing the distances as described in the previous study (11). The stability of branches was assessed after bootstrapping replicates (*n* = 1,000).

### Drug resistance analysis of *S. Aureus* bovine milk isolates *via* Kirby Bauer technique

The disk diffusion method was performed to examine the antimicrobial sensitivity profile of *S. aureus* isolated from bovine milk samples, according to the criteria introduced by the Clinical and Laboratory Standards Institute (CLSI, 2021). Plates with LB agar were used with 18 commercial antimicrobial agents (Hangzhou Binhe Microorganism Reagent Co., Ltd., China), namely penicillin G (10 μg, Catalog # C001), cefoxitin (30 μg, Catalog # C058), SXT (1.25 μg, Catalog # C027), ciprofloxacin (5 μg, Catalog # C045), norfloxacin (10 μg, Catalog # C033), vancomycin (30 μg, Catalog # C030), polymyxin B (300 μg, Catalog # C025), levofloxacin (5 μg, Catalog # C066), macrodantin (300 μg, Catalog # C028), tetracycline (30 μg, Catalog # 021), chloramphenicol (30 μg, Catalog # 022), oxacillin (1 μg, Catalog # C004), phosphonomycin (200 μg, Catalog # C092), clindamycin (2 μg, Catalog # C093), minocycline (30 μg, Catalog # C046), clarithromycin (15 μg, Catalog # C063), erythromycin (15 μg, Catalog # C023), and spectinomycin (30 μg, Catalog # C036), for antimicrobial sensitivity detection. Each examination was performed three times. Laboratory stored *S. aureus* (ATCC 25923) and *E. coli* (ATCC 25922) were employed as positive and negative control strains, respectively.

### Drug-resistant genes and virulence genes amplification

Genomic DNA from drug-resistant *S. aureus* isolates was extracted using a commercial bacterial genomic DNA extraction kit (Solarbio life sciences, China) as previous studies described (1). The resistant genes and virulence genes were amplified by piloting specific primers (Table 1) as described in a previous study (11–59). The 25 μl PCR reaction mixture contains 2 μl DNA template, 1 μl of forward and reverse primers, respectively, 12.5 μl of the Taq PCR Master Mix (Sangon Biotech, China), and 8.5 μl of distilled water. The PCR amplification contained 30 PCR cycles with 95°C for 30 s, Tm for 45 s, and 72°C for 60 s in each cycle after an initial hot start at 95°C for 5 min and ending with 72°C for 10 min. After that, all the PCR products were examined through 1.2% agarose gel electrophoresis.

**Table 1:** The primers information of drug-resistant genes and virulence genes.

**Target**	**Primer sequence (5^′^-3^′^)**	**Tm (°C)**	**Amplicon size (bp)**	**Reference**
*Nuc*	AGTTCAGCAAATGCATCACA	56	400	(26)
	TAGCCAAGCCTTGACGAACT			
*Pvl*	ATCATTAGGTAAAATGTCTGGACATGATCCA	57	433	(11)
	GCATCAASTGTATTGGATAGCAAAAGC			
*fnbA*	GTGAAGTTTTAGAAGGTGGAAAGATTAG	54	643	(27)
	GCTCTTGTAAGACCATTTTTCTTCAC			
*fnbB*	GTAACAGCTAATGGTCGAATTGATACT	54	524	(27)
	CAAGTTCGATAGGAGTACTATGTTC			
*Hla*	CTGATTACTATCCAAGAAATTCGATTG	52	209	(27)
	CTTTCCAGCCTACTTTTTTATCAGT			
*Sea*	GAAAAAAGTCTGAATTGCAGGGAACA	55	560	(27)
	CAAATAAATCGTAATTAACCGAAGGTTC			
*Seb*	ATTCTATTAAGGACACTAAGTTAGGGA	57	404	(27)
	ATCCCGTTTCATAAGGCGAGT			
*Eta*	CGCTGCGGACATTCCTACATGG	57	676	(26)
	TACATGCCCGCCACTTGCTTGT			
*Etb*	CAGATAAAGAGCTTTATACACACATTAC	52	612	(27)
	AGTGAACTTATCTTTCTATTGAAAAACACTC			
*tsst*-*1*	TTCACTATTTGTAAAAGTGTCAGACCCACT	57	180	(27)
	TACTAATGAATTTTTTTATCGTAAGCCCTT			
*Clfa*	ATTGGCGTGGCTTCAGTGCT	60	292	(27)
	CGTTTCTTCCGTAGTTGCATTTG			
*mecA*	ATGAAAAAGATAAAAATTGTTCCAC	49	1435	(28)
	ATTTCTTACTGCCTAATTCGAG			
*mecB*	TTAACATATACACCCGCTTG	51	527	(28)
	TAAAGTTCATTAGGCACCTCC			
*mecC*	AAGTTAATCAAAAATGGGTWCAGC	51	1 309	(28)
	ACGTCTTTAACATTAATYGCCA			
*mecD*	TCCTTTAGCGATAGATGGTGAA	53	834	(28)
	CTCCCATCTTTTCTCCATCCT			
*blaZ*	TAAGAGATTTGCCTATGCTT	49	377	(29)
	TTAAAGTCTTACCGAAAGCAG			
*ermA*	GTTCAAGAACAATCAATACAGAG	51	421	(30)
	GGATCAGGAAAAGGACATTTTAC			
*ermB*	CCGTTTACGAAATTGGAACAGGTAAAGGGC	61	359	(30)
	GAATCGAGACTTGAGTGTGC			
*ermC*	GCTAATATTGTTTAAATCGTCAATTCC	56	572	(30)
	GGCTCAGGAAAAGGGCATTTTAC			
*aphA*	CCAAGAGCAATAAGGGCATACC	56	382	(31)
	ACCCTCAAAAACTGTTGTTGC			
*acc(6')-aph(2″)*	GGAAGCAGAGTTCAGCCATG	56	675	(31)
	TGCCTGCATATTCAAACAGC			
*ant(4')-Ia*	ACAGCCGGTATAAAGGGACCACC	61	266	(31)
	AAAATCATACAGCTCGCGCGGATC			
*fusB*	ATTCAATCGGAAACCTATATGATA	48	292	(29)
	TTATATATTTCCGATTTGATGCAAG			
*ileS*	TATATTATGCGATGGAAGGTTGG	51	458	(29)
	AATAAAATCAGCTGGAAAGTGTTG			
*rpoB*	AGTCTATCACACCTCAACAA	53	702	(29)
	TAATAGCCGCACCAGAATCA			

## Results

### Bacteria isolation and identification

A total of 24.8% (31/125) of bacteria isolates were isolated by a Mannitol salt agar plate from clinically positive samples. Gram staining showed Gram-positive purple grape-globose bacteria, and biochemical reactions revealed positive results of N-acetyl-glucosamine, trehalose, sucrose, urea, mannose, maltose, N-mannitol, and lactose, respectively, while negative results were observed for xylose and nitrate reduction. The agarose gel electrophoresis indicated ~1,500 bp bands of 16 s rRNA genes of bacteria, and then those positive PCR bands were purified, sequenced, and standard nucleotide blasting was performed with NCBI databases (https://blast.ncbi.nlm.nih.gov/Blast.cgi?PROGRAM=blastn&PAGE_TYPE=BlastSearch&LINK_LOC=blasthome). The confirmed 16 s rRNA gene of *S. aureus* was deposited into the NCBI database with accession number ON138912-ON138914.

### Multiple sequence alignment and phylogenetic analysis of 16 s rRNA gene of *S. Aureus* with available reference genes

**Figure 1:**
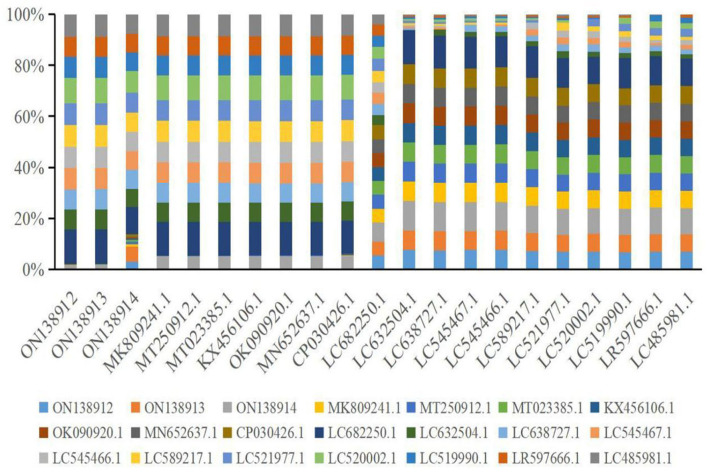
Multiple alignments analysis of 16s rRNA gene of *Staphylococcus aureus (S. aureus)* with reference strains.

**Figure 2:**
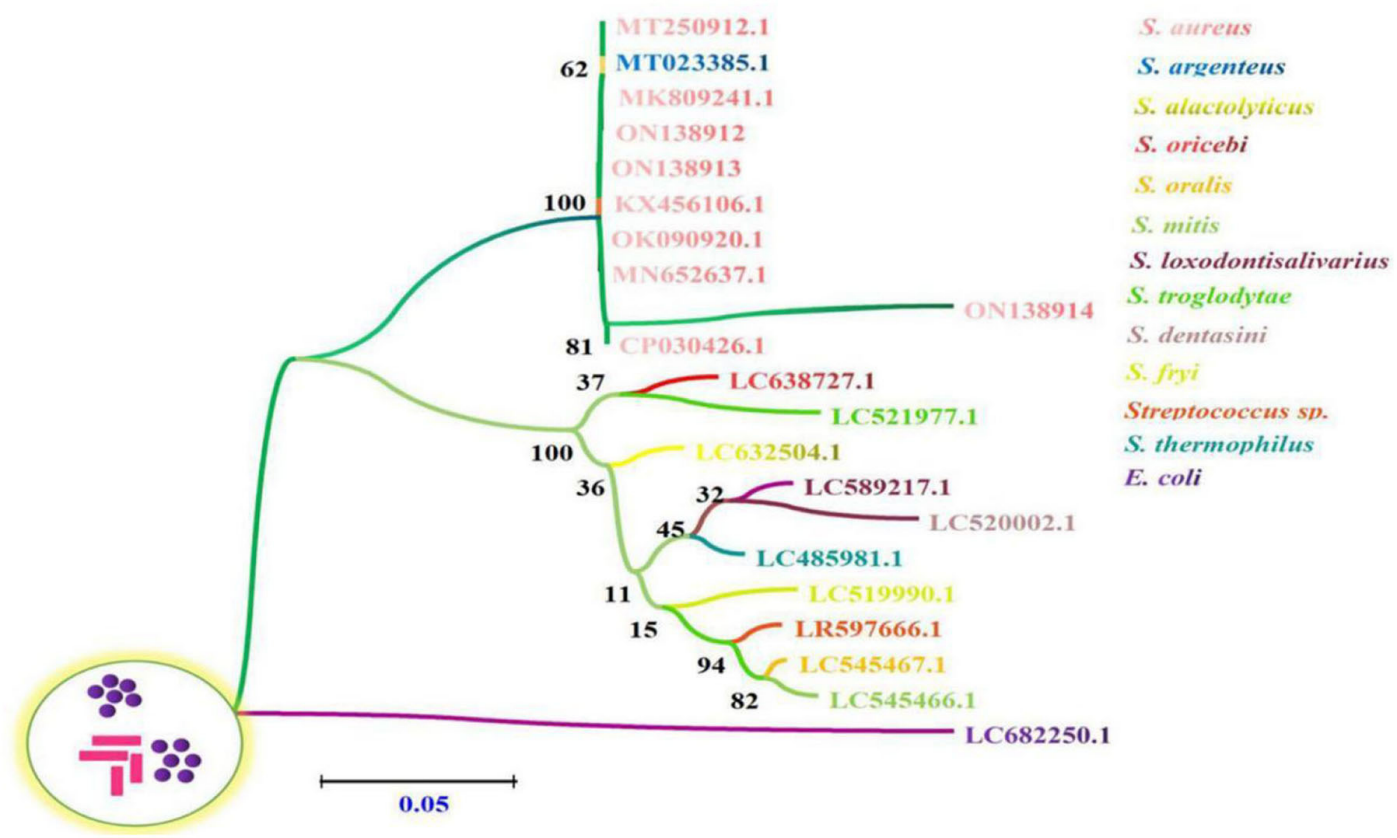
The phylogenetic relationships of 16s rRNA gene between *S. aureus* sequences derived from bovine milk samples and reference sequences by employing a Neighbor-Joining (NJ) method *via* Kimura two-parameter analysis. The number of nodes indicates the bootstrap values. Bootstrap values >50% from 1,000 replicates are shown on the nodes.

Multiple sequence alignment analysis found that the current isolates ON138912, ON138913, and ON138914 were highly similar (96.9–100.0%) to MK809241.1, MT250912.1, MT023385.1, KX456106.1, OK090920.1, MN652637.1, and CP030426.1, especially ON138912 and ON138913 isolates (Figure 1). Phylogenetic analysis demonstrated that ON138912 and ON138913 clade with MK809241.1 were isolated from food in China, whereas ON138914 clade with CP030426.1 was isolated from a person from the USA (Figure 2).

### Drug resistance analysis, drug-resistant genes, and virulence genes amplification analysis of *S. Aureus*

**Figure 3:**
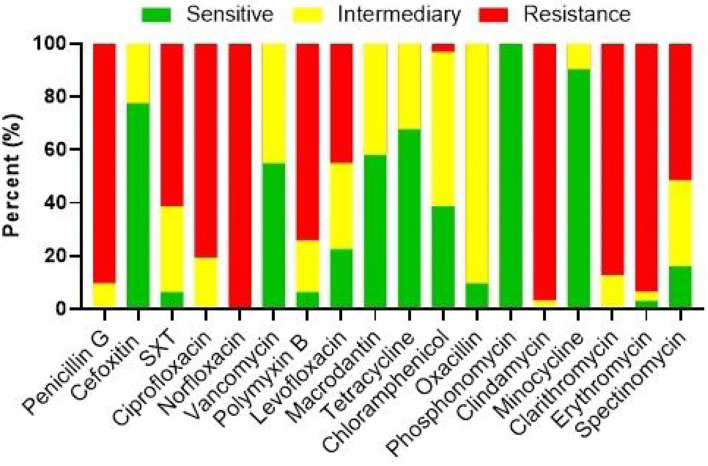
The antibiotic resistance analysis of *S. aureus*.

**Figure 4:**
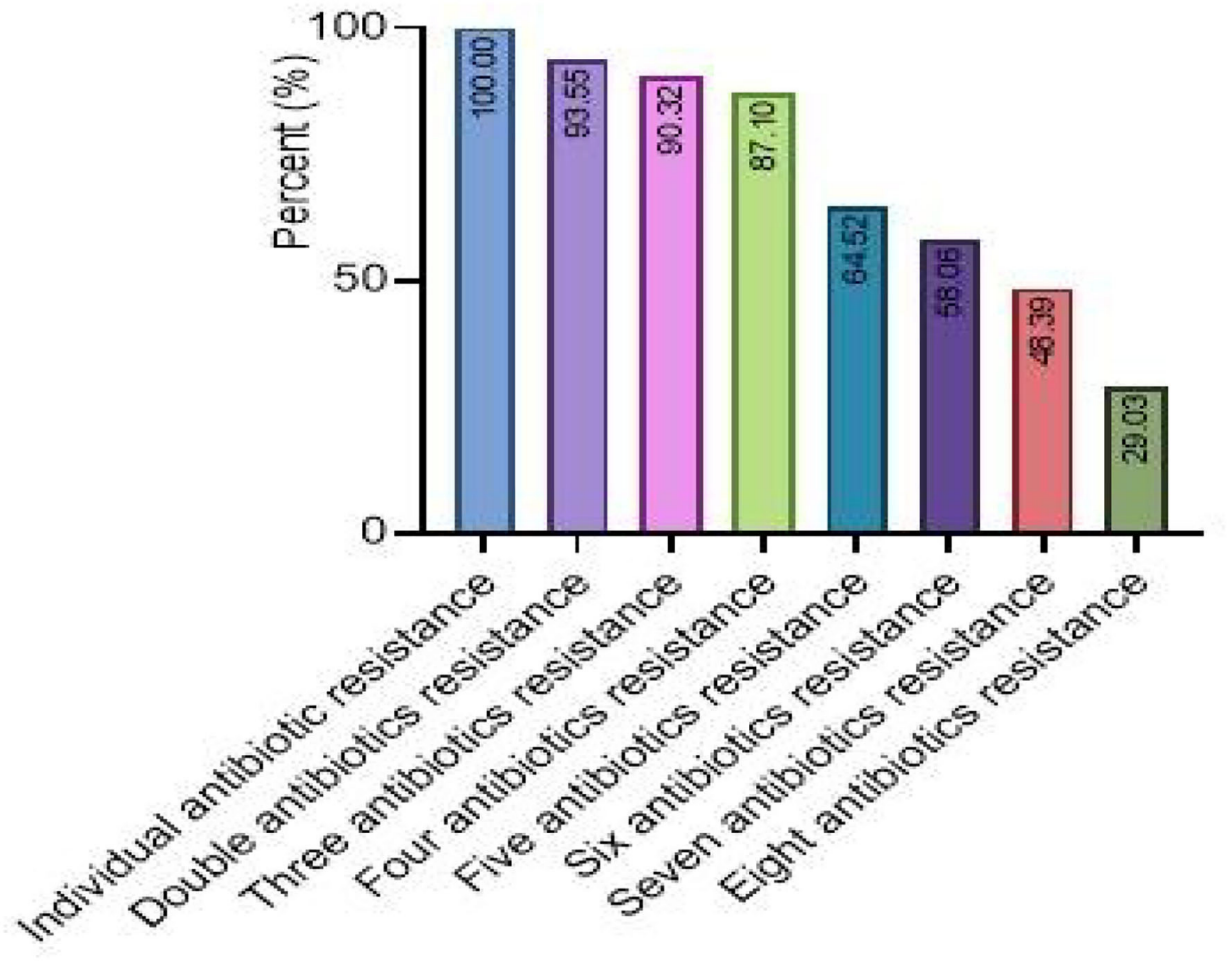
The multi-drug resistance analysis of *S. aureus*.

**Figure 5:**
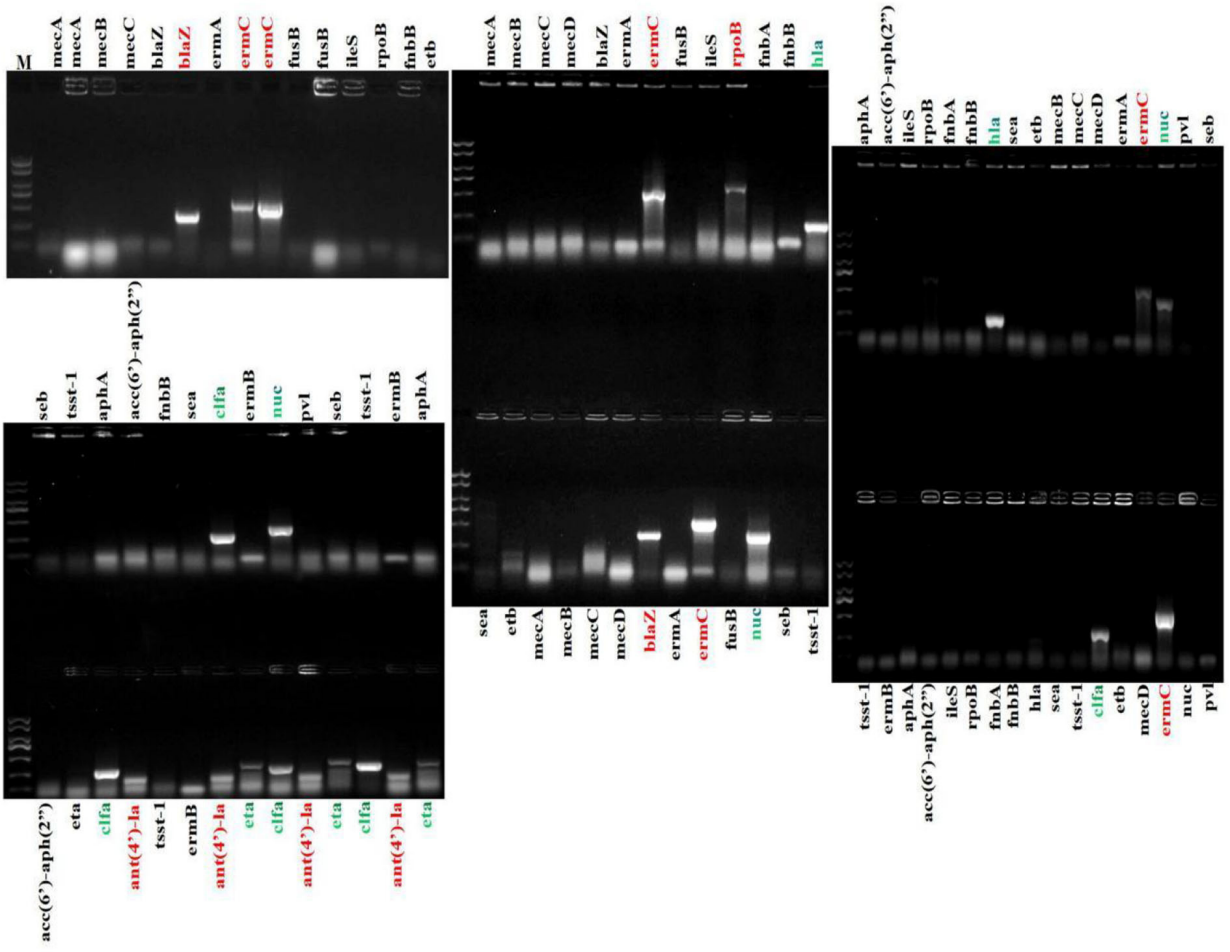
Amplification of drug resistance genes and virulence genes of *S. aureus*. Marker ladder: 2,000, 1,500, 1,000, 750, 500, 250, and 100 bp.

The present bacterial isolates were detected resistant to 11 antibiotics, namely penicillin G, SXT, ciprofloxacin, norfloxacin, polymyxin B, levofloxacin, chloramphenicol, clindamycin, clarithromycin, erythromycin, and spectinomycin (Figure 3), with drug resistance rate ranging from 3.22 (1/31) to 100% (31/31). In these drug resistance *S. aureus*, double antibiotic resistance to eight antibiotics was examined with the prevalence of 29.03 (9/31) to 93.55% (29/31) (Figure 4). Then, according to the antibiotic-resistant results, the amplification of the 14 commonly known drug-resistant genes (blaZ, ermC, rpoB, and ant(4')-la) was successfully amplified (Figure 5). The virulence genes of hla, nuc, clfa, and eta were found in the current *S. aureus* bacteria (Figure 5).

## Discussion

Mastitis is one of the major problems that severely affect the dairy sector globally (1). It has a prominent deteriorated impact on milk production, milk quality, and ruminant health and welfare with considerable economic damage (11). *S. aureus* is recognized as the third most important worldwide food-borne pathogen which produces bacterial enterotoxins (32). The polluted milk products were frequently mixed up with the *S. aureus* contamination (33). Milk and its related products are important macro-nutrients for consumers, which makes them vulnerable to pathogens contamination (34–40). With the emergence of anti-microbial *S. aureus* increasing day by day, it is important to regularly perform the monitoring and examination of the prevalence, and the molecular characterization of *S. aureus* in mastitis suspected milk samples.

In the current study, *S. aureus* was isolated from milk samples, which was identified by Gram staining and biochemical reactions. This was then confirmed by the amplification of 16 s rRNA genes of bacteria, sequencing, and blasting results. Multiple sequence alignment analysis found that the current isolates (ON138912, ON138913, and ON138914) were highly similar (96.9–100.0%) to previous isolates (MK809241.1, MT250912.1, MT023385.1, KX456106.1, OK090920.1, MN652637.1, and CP030426.1). Phylogenetic analysis demonstrated that ON138912 and ON138913 clade with MK809241.1 were isolated from food in China, whereas ON138914 clade with CP030426.1 was isolated from a person from the United States of America.

The prevalence of *S. aureus* isolated from milk samples was 24.8% (31/125), which is in line with the prevalence of *S. aureus* in cow bulk tank milk samples in the region of Balikesir in Turkey (28%) (4) and milk samples obtained from acute mastitis cases in Jordan (23). The prevalence was lower than in bovine mastitis milk samples in the US (46.6%), Indonesia (57%), and Bangladesh (72.7%) (11, 32, 41), but higher than in milk samples obtained from culled cows in Jordan (13.7%) (23), milk samples in cows in Ethiopia (12.5%) (10), and composite milk samples in dairy cows in South Africa (42). This variability between different study results could be attributed to the differences in geographical, study population, farm hygienic and management practices (such as breeds, farm size, the practice of hand milking, and absence of dry cow therapy), milking methods, and instruments employed (11, 42).

Herein, the four virulence genes of hla, nuc, clfa, and eta were examined in the current *S. aureus* bacteria, which differed from the six genes named sea, seb, seg, sei, selp, and tsst1 in a previous study in *S. aureus* isolated from tank milk samples in Turkey (43), and twenty-two genes (aur, splA, hlgC, hlb, lukE, seg, sei, sem, sen, seo, seu, seb, sec, sed, seh, lukD, sek, splB, hlgA, sel, seq, and hlgB) from milk samples in the US (32). Exfoliative toxin (eta) genes were rarely detected in *S. aureus* isolated from mastitis positive dairy animals. The present study and previous studies found eta gene in *S. aureus* from cows in Belgium and Poland (44, 45), which illustrated that the bovine *S. aureus* evolutionary precursors clones had an important relationship with human *S. aureus* clones (46). Hemolysins of staphylococcal were considered pivotal factors related to bacterial invasion and evading host immunity (47). Of these, alpha hemolysin (hla) toxin is commonly accepted as the most emphasized and characterized virulence factor in *S. aureus* (48). ClfA is one of the important *S. aureus* genes for adhesion to extracellular matrix proteins to colonize and establish infections (48). *S. aureus* nuclease (nuc) is highly related to the trapping of bacteria biofilm (49). Those four virulence genes examined in *S. aureus* isolated from milk samples may combine to make the bacterium a versatile pathogen (50).

For the prevention and control of mastitis in cattle, a wide range of antimicrobials are commonly used (51), however inappropriate and excessive use of antibiotics leads to resistant bacteria. In the present results, a high antibiotic resistance rate was found, which is in line with previous studies of drug resistant *S. aureus* in South Africa and Turkey (4, 42). The resistance of *S. aureus* to penicillin G, SXT, ciprofloxacin, norfloxacin, polymyxin B, clindamycin, clarithromycin, and erythromycin was significant, which indicates that it is unsuitable to employ those antibiotics in this region. In current findings, blaZ, ermC, rpoB, and ant(4')-Ia are successfully amplified, which is not in agreement with previous studies in which *S. aureus* harboring norA, aph (3′)-Ia, mecA, ant (6)-la, aph (3′) III, mph(C), msr(A), norA, and blaZ (32). β-lactamases (blaZ) is one of the important enzymes which resist penicillin (48, 52); the current results are in line with a previous study about blaZ in *S. aureus* (50). The drugs (clarithromycin, erythromycin, spectinomycin, clindamycin, and streptogramin B) resistant gene ermC, which is detected in *S. aureus* from milk samples in the present study are commonly found in staphylococci from mastitis positive animals in Brazil (53, 54). A gene point mutation associated with resistance against rifampicin of rpoB is likely found in *S. aureus* (55). Ant(4′)Ia is one of the important enzymes causing aminoglycoside resistance in *S. aureus* (56). Those four drug resistance genes found in *S. aureus* may further confirm multiple drug resistance bacteria in the study area.

Infected cattle at dairy farms may transmit pathogens to other animals and farm workers who have close contact with cows (57). The bacteria from raw milk may also cause food-poisoning infection through the ingestion of *S. aureus* contaminated milk (34). Therefore, the multi-resistance of *S. aureus* isolates found in cow milk samples is an important concern for animals and public health. The prevention and control strategy of mastitis caused by bacteria *S. aureus* could be achieved by pathogens isolation and characterization, infected animals' segregation, dry cow therapy, and timely treatment of infected cases (11). *S. aureus* is a potential reservoir for toxins that bring harmful effects on human health (26, 27, 45, 58). Therefore, there is a need to establish countermeasures to promote careful utilization of antibiotics to reduce drug resistance to *S. aureus* development in dairy cattle.

## Conclusion

The current study isolated *S. aureus* from milk samples and revealed its drug-resistant situation, drug-resistant genes, and virulence genes. So, regular monitoring of *S. aureus* in milk samples from dairy cows may contribute to the prevention and treatment of public health concerns causing bacteria in this region.

## Limitations of study

The mastitis bovine milk samples were collected from a dairy farm, which is the limitation of this study. Therefore, it is recommended to conduct this study throughout the province for a better indication of multi-drug-resistant *Staphylococcus aureus*.

## Data availability statement

The datasets presented in this study can be found in online repositories. The names of the repository/repositories and accession number(s) can be found below: https://www.ncbi.nlm.nih.gov/, ON138912.1, ON138913.1, ON138914.1.

## Ethics statement

All the procedures of the study were performed under the approval of Laboratory Animals Research Center of Hebei and Anhui province in P. R. China, and the Ethics Committee of Hebei Agricultural University.

## Author contributions

JL: methodology and writing original draft. CB and ZH: supervision and visualization. XW, KM, and JQ: reagents, materials, and analysis of tools. JL, XW, KM, and FA: writing review and editing. CB, XW, and ZH: conceptualization, funding, and resources. All authors contributed to the article and approved the submitted version.

## Funding

The current research was supported by the establishment and application of a new mode for prevention and control of mixed infection of main animal diseases, Shandong Province agricultural major application technology innovation project (SD2019XM007); the evaluation test of animal disease detection reagents of Hangzhou Bori Technology Co., Ltd. (LYDX-BIOER-202011); the development and application of animal disease monitoring and early warning system (LYDX-SPRING-202107).

## Conflict of interest

The authors declare that the research was conducted in the absence of any commercial or financial relationships that could be construed as a potential conflict of interest.

## Publisher's note

All claims expressed in this article are solely those of the authors and do not necessarily represent those of their affiliated organizations, or those of the publisher, the editors and the reviewers. Any product that may be evaluated in this article, or claim that may be made by its manufacturer, is not guaranteed or endorsed by the publisher.
