# Supplementary figures and images for: Molecular characterization of multi-drug-resistant Staphylococcus aureus in mastitis bovine milk from a dairy farm in Anhui, China
Source: Front Vet Sci. 2022 Aug 22;9:966533. doi: 10.3389/fvets.2022.966533 (PMC9443526; doi:10.3389/fvets.2022.966533)

**FIGURE S1:** The geographical map of Anhui province.

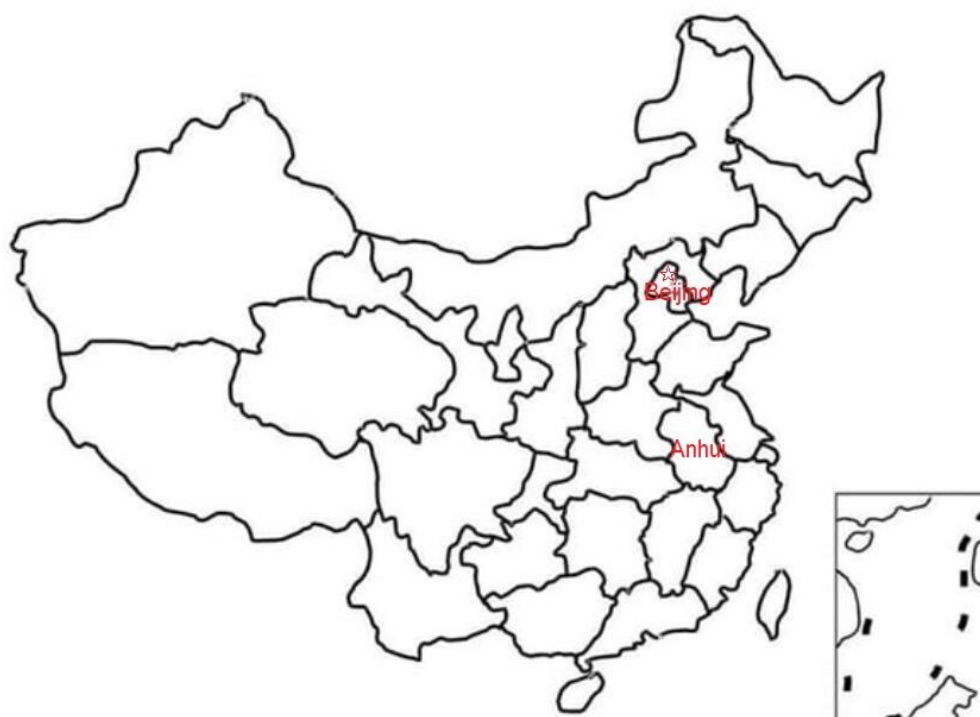

Supplement: Supplementary file 1 [file Image_1.pdf]
